# Immune Modulation by Antigenic Peptides and Antigenic Peptide Conjugates for Treatment of Multiple Sclerosis

**DOI:** 10.18103/mra.v10i5.2804

**Published:** 2022-06-01

**Authors:** Rucha Mahadik, Paul Kiptoo, Tom Tolbert, Teruna J. Siahaan

**Affiliations:** 1 Department of Pharmaceutical Chemistry, School of Pharmacy, The University of Kansas, 2093 Constant Avenue, Lawrence, KS 66047; 2 Sanofi Pharmaceuticals, 2 1 The Mp

**Keywords:** antigenic peptides, bifunctional peptide inhibitor (BPI), I-domain antigen conjugates (IDAC), Fc-BPI, peptide polymer conjugates, multiple sclerosis (MS), EAE, immunological synapse, T cells, antigen-presenting cells (APCs), Th1, Th17, Treg

## Abstract

The immune system defends our body by fighting infection from pathogens utilizing both the innate and adaptive immune responses. The innate immune response is generated rapidly as the first line of defense. It is followed by the adaptive immune response that selectively targets infected cells. The adaptive immune response is generated more slowly, but selectively, by targeting a wide range of foreign particles (i.e., viruses or bacteria) or molecules that enter the body, known as antigens. Autoimmune diseases are the results of immune system glitches, where the body’s adaptive system recognizes self-antigens as foreign. Thus, the host immune system attacks the self-tissues or organs with a high level of inflammation and causes debilitation in patients. Many current treatments for autoimmune diseases (i.e., multiple sclerosis (MS), rheumatoid arthritis (RA)) have been effective but lead to adverse side effects due to general immune system suppression, which makes patients vulnerable to opportunistic infections. To counter these negative effects, many different avenues of antigen specific treatments are being developed to selectively target the autoreactive immune cells for a specific self-antigen or set of self-antigens while not compromising the general immune system. These approaches include soluble antigenic peptides, bifunctional peptide inhibitors (BPI) including IDAC and Fc-BPI, polymer conjugates, and peptide-drug conjugates. Here, various antigen-specific methods of potential treatments, their efficacy, and limitations will be discussed along with the potential mechanisms of action.

## Introduction

**Figure 1.**
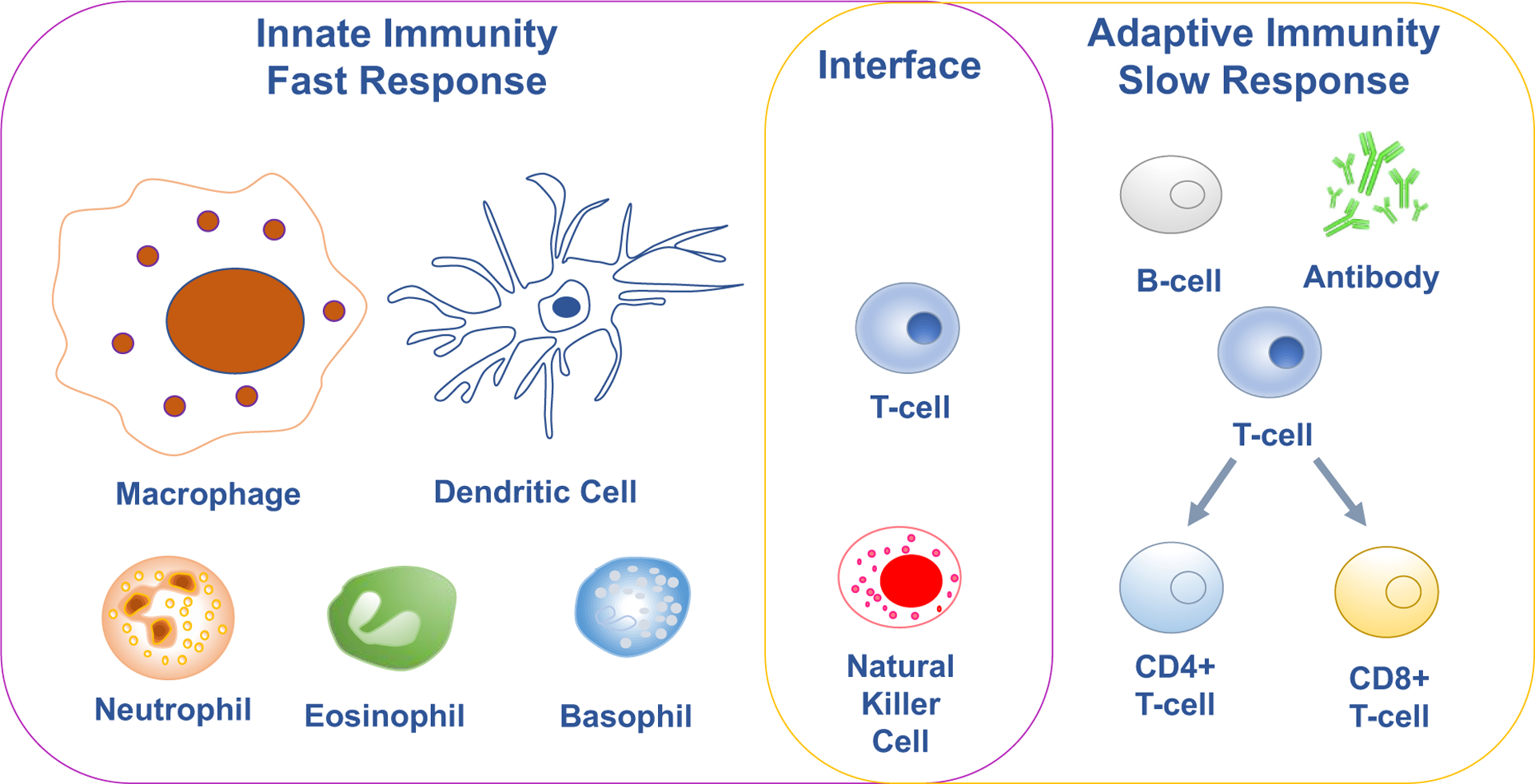
The immune system has innate and adaptive immunity. The innate immune response is a fast response generated by macrophages, dendritic cells, neutrophils, eosinophils, basophils and natural killer cells. The adaptive immune response is a slow immune response generated by B cells and T cells. B cells are responsible to produce antibodies that recognize the pathogen for the humoral response. Antigen-specific T cells such as CD4^+^ and CD8^+^ T cells are generated as the cell-mediated response to pathogen infection. T cells and natural killer cells are at the interface of innate and the adaptive immunity.

The body’s immune system is a complex molecular and cellular system that protects the body from insult by a wide variety of pathogens that enter the host. Innate immune responses utilize physical barriers and surface pattern recognition of pathogens to generate immune responses using pattern recognition receptors (PRR) on the surfaces of immune cells.^1^ The innate immune system reacts fast to infection as the first line of response by identifying pathogen associated molecular patterns (PAMPs), such as unusual mannose containing oligosaccharides on bacteria and fungi. Innate immune responses directed at PAMP containing pathogens include activation of the complement system, release of chemical mediators and cytokines to induce inflammation, and recruitment of white blood cells to sites of infection (Figure 1). Cells that make up the innate immune system include dendritic cells, macrophages, neutrophils, basophils, eosinophils and natural killer cells (Figure 1).^1^ While prompt to respond to an infection, the innate immune system has no memory, meaning that it will launch the same response of engulfing or eliminating if it encounters the same pathogen later. A second and slower response, called the adaptive immune response, is more selective and targeted compared to the innate immune response, and is initiated through a process of antigen presentation and recognition of non-self antigens.^1^ In the adaptive immune response, specific cells (i.e., B and T cells) are activated to target pathogens or cells infected with pathogen. For this response, antigen specific B and T cells must be activated and then proliferate to produce enough highly specific cells to respond against the pathogen. It should be noted that the maturation process of B and T cell receptors requires time. This means that while highly effective, it takes time for the adaptive immune system to mount an attack to fight off the pathogens. Though it takes longer, the adaptive immune system develops memory lymphocytes that can help launch a faster response if it encounters the same pathogen again.

Generally, when a pathogen attempts to invade the host, it will first encounter the innate immune system.^1^ This includes physical barriers such as epithelial layers and/or mucosal layers, or cells with PRR that recognize non-host pathogen patterns and bind to them. The cells of the innate immune response will activate cells from the adaptive immune response, which are the antigen-specific B and T cells.^1, 2^ The adaptive immune response is broken down into humoral and cellular immune responses. The humoral response is initiated by activation of B cells for antibody production via plasma cells. The generated antibodies can target the pathogen for neutralization and opsonization followed by initiating the complement system to stop proliferation of the pathogen.^3^ B cell activation is dependent on both antigen presentation to B cell receptors and the assistance of a helper T cell (Th), a type of CD4^+^ cell. Cell-mediated immunity activates CD8^+^ and CD4^+^ T cells that recognize specific antigens presented on the surface of cells via MHC-I and MHC-II complexes.^2^ This recognition allows for the T cells to identify the infected cells followed by recruitment of other immune cells for eliminating the infected cells.^2^ At the same time, both B and T cells create memory cells that allow for a faster adaptive immune response to that antigen if there is a reinfection.

The wide-range of antigen-specific lymphocytes allows for the immune system to specifically target all pathogens. Having a wide range of antigen receptors means that lymphocytes can potentially be specific to protein regions or peptide sequences native to the body, or self-antigens. Theoretically, lymphocytes that recognize self-antigens have been eliminated during development. In addition, there are multiple tolerance mechanisms present to remove autoreactive lymphocytes. However, autoreactive lymphocytes can evade the tolerance mechanisms. The onset of autoimmune disease can be triggered by the activation of these autoreactive lymphocytes; this activation has been observed in rheumatoid arthritis (RA), multiple sclerosis (MS), and type-1 diabetes (T1D).^4^ Autoimmune diseases can be chronic and life-threatening; these patients need life-long therapy.

Disease-modifying therapies (DMTs), anti-inflammatories, and immunosuppressant drugs have been prescribed to patients for treatments of autoimmune diseases.^4, 5^ Anti-inflammatories are aimed at reducing side effects of the disease for short-term treatment.^6^ Because autoimmune diseases are a result of immune cells attacking self-tissues or organs, the immunosuppressant drugs are used long-term to lessen the activity of immune cells. However, these drugs are not cell specific; the use of these drugs can lead to global immunosuppression in the body.^5, 7^ As a result, patients are vulnerable to opportunistic infections and cancers.^5^ DMTs treat autoimmune diseases by interfering with immune cell activity in general.^5^ As an alternative, antigen-specific immunotherapies have been developed to target only rogue cells that cause the autoimmune disease and suppress their proliferation as well as disease progression. Therefore, various new developments of antigen-specific treatments and their potential mechanisms of action will be discussed here.

## 2. Onset of Autoimmune Diseases

**Figure 2.**
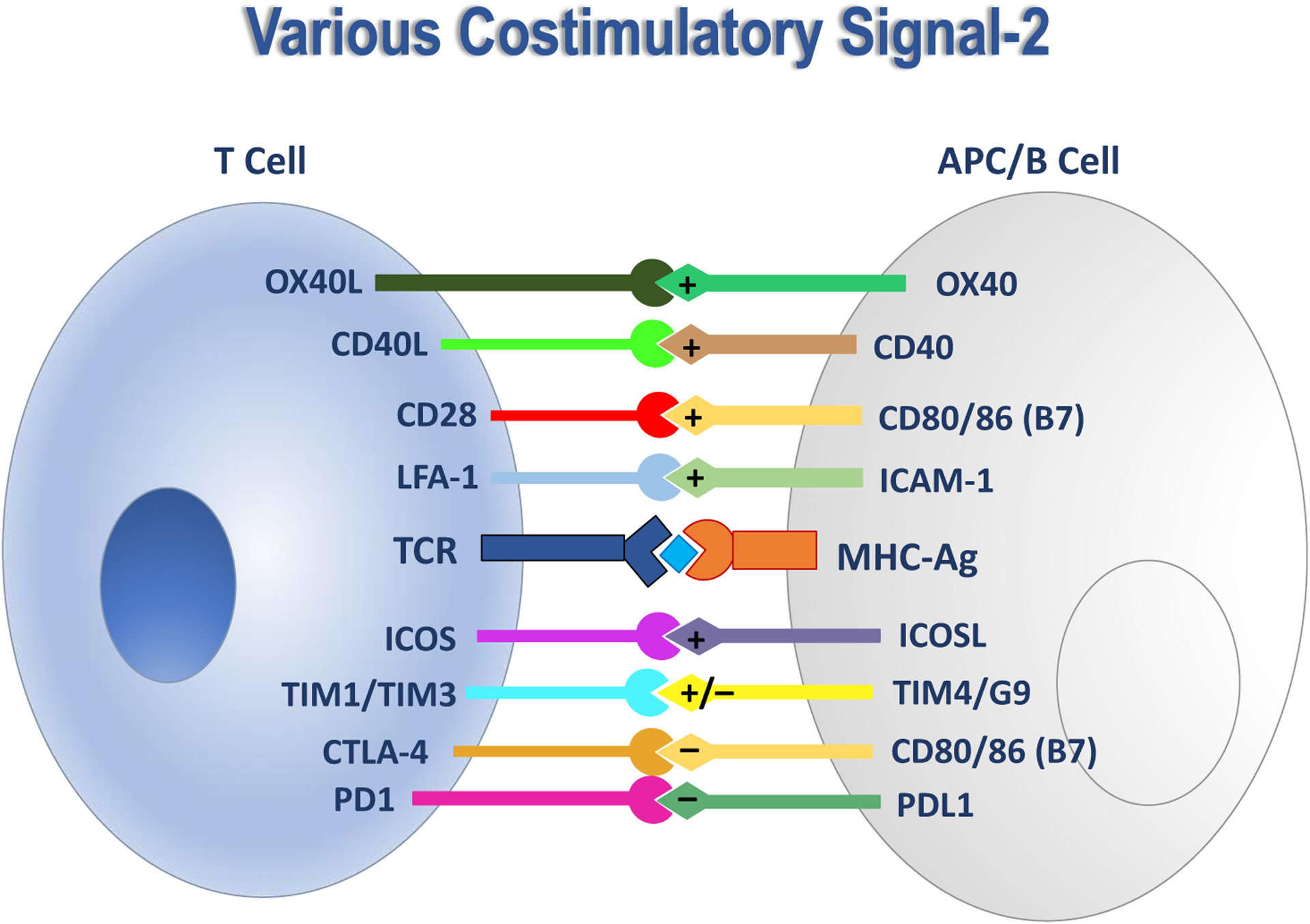
There are various signaling proteins on the surface of antigen presenting cells (APCs) and T cells. The first signal, Signal-1, is generated by interactions between Ag-MHC-II complex and TCR to produce TCR/Ag-MHC-II complex. The costimulatory signal or Signal-2 is divided into positive and negative signals. The positive costimulatory signal can be generated by OX40L/OX40, CD40L/CD40, CD28/CD80, CD28/CD86, LFA-1/ICAM-1, or ICOS/ICOSL. The negative signal can be stimulated by CTLA-4/CD80, CTLA-4/CD86, or PD1/PDL1.

The trigger for the onset of autoimmune diseases is the recognition of self-antigens by B and T cells, followed by the activation and proliferation of T cells specific to the self-antigens. T cell activation is a product of naïve T cell interactions with dendritic cells and other antigen presenting cells (APCs). The antigen is engulfed and processed by APCs and subsequently antigen fragments are presented as antigen-MHC-II complexes (Ag-MHC-II) on the surface of APC. Then, T-cell antigen receptors (TCRs) bind with Ag-MHC-II as a cluster of Ag-MHC-II/TCR complexes (i.e., Signal-1). To activate T cells, Signal-1 is accompanied by Signal-2 generated by a cluster of complexes of costimulatory molecules at the APC/T cell interface (Figure 2).^8^

**Figure 3.**
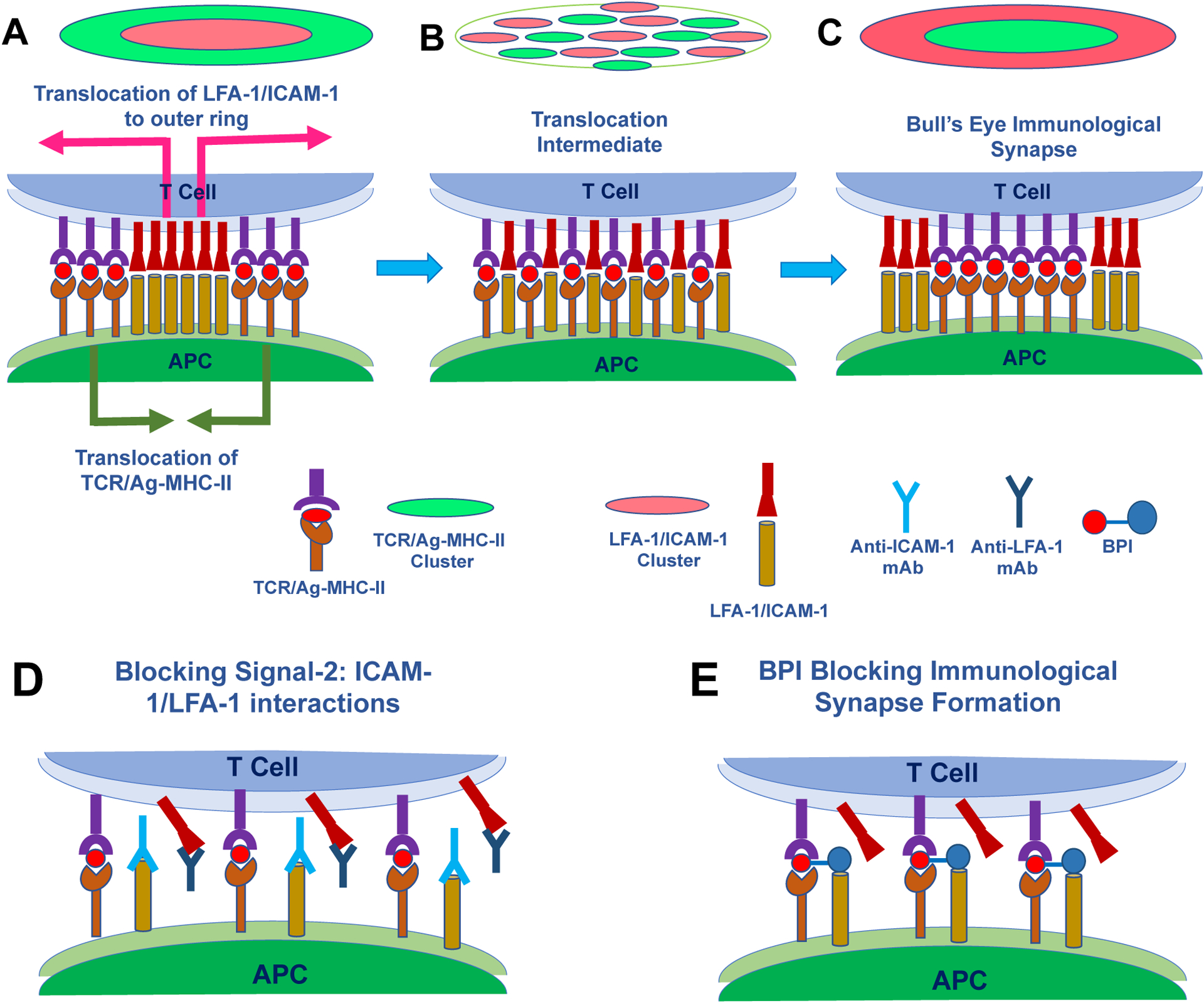
The mechanism of immunological synapse (IS) formation at the interface of T cell and APC to activate the proliferation of antigen-specific inflammatory T cells (i.e., Th1 and Th17). **(A)** The initial interaction between the T cell and APC is mediated by LFA-1/ICAM-1 complexes that form a cluster at the center (Red) and TCR/Ag-MHC-II complexes to form a cluster at the peripheral region (Green). LFA-1/ICAM-1 complexes translocate from the center to the periphery and at the same time TCR/Ag-MHC-II complexes translocate into the center. **(B)** The intermediate stage during translocation between LFA-1/ICAM-1 complexes and TCR/Ag-MHC-II complexes in which the formation of the IS is not completed yet. **(C)** The formation of the IS is when a cluster of TCR/Ag-MHC-II complexes in the center (Green) as central supramolecular activation complex (cSMAC) and a cluster of LFA-1/ICAM-1 complexes in the peripheral region as peripheral supramolecular activation complex (pSMAC). **(D)** Blocking Signal-2 or ICAM-1/LFA-1 interactions using anti-ICAM-1 and/or anti-LFA-1 mAbs in the presence of Signal-1 disrupts the formation of IS to induce tolerance by suppressing the proliferation of inflammatory Th1 and Th17 cells. **(E)** The proposed mechanism of activity of BPI molecule in suppressing inflammatory T cell activation and proliferation from naïve T cells. BPI molecules simultaneously bind to MHC-II and ICAM-1 on APC. Interaction of the T cell with the APC generates TCR/Ag-MHC-II complexes but binding of cell adhesion fragment (LABL peptide) to ICAM-1 blocks the LFA-1/ICAM-1 interactions. As a result, the BPI inhibits the formation of the IS to stop proliferation of inflammatory Th1 and Th17 cells.

Besides Signal-1, T cells are also activated during the inflammatory response by Signal-2 through connectivity of co-stimulatory molecules (Figure 3).^6, 9^ The interactions of both signals (Signal-1 and Signal-2) form the immunological synapse (IS), which involves positional translocation between Signal-1 and Signal-2 complexes. The Signal1/Signal-2 translocation was demonstrated using peptide-MHC-II/TCR and ICAM-1/LFA-1 interactions, respectively, using T cells *in vitro*.^10–12^ To study the IS, planar membranes were studded with fluorescently labeled peptide:MHC-II as a component of Signal-1 and ICAM-1 as a component of Signal-2 on the APC. Upon addition of the T cell, LFA-1 molecules on T cell bind to ICAM-1 molecules on planar membranes to form a cluster of Signal-2 in the center while a cluster of peptide:MHC-II/TCR complexes as Signal-1 form in the periphery (Figure 3A).^10^ After 5 min, the ICAM-1/LFA-1 complexes translocate from the center to peripheral to make peripheral supramolecular activation complex (pSMAC) through an intermediate (Figure 3B), while peptide:MHC-II/TCR complexes translocate from the peripheral to the center to make central supramolecular activation complex (cSMAC). Finally, both clusters formed a “Bull’s Eye” structure called the IS (Figure 3C).

The IS formation stimulates the differentiation of naïve T cells to antigen-specific CD4^+^ T cells (Th1 and Th17 cells) that induce production of inflammatory cytokines such as TNF-α, IFNγ, and IL-17 in delayed-type hypersensitivity (DTH) and autoimmune diseases. There are various positive and negative costimulatory signal molecules (Figure 2). The positive costimulatory signals induce the activation of inflammatory T cells; in contrast, the negative costimulatory signals suppress the activation of inflammatory T cells.^13–15^ The positive signal (Signal-2) can be generated by CD28/CD80 (B7-1), CD28/CD86 (B7-2) or LFA-1/ICAM-1 interactions^6, 15–17^ while CTLA-4/B7-1 and CTLA-4-B7-2 interactions produce negative signals.^16, 17^ Blocking the positive costimulatory signal via CD28 induces T cell anergy and immunotolerance by expanding the regulatory T cell (Treg) population.^15, 18–22^ The CD28/B7 signal has a distinct effect from the LFA-1/ICAM-1 signal in the differentiation of CD4^+^ T cells to generate Th1 and Th2 effector phenotypes.^23–25^ Th2 cells mediate the hypersensitivity reaction in allergy and asthma by producing IL-4, IL-5, IL-10, and IL-13 cytokines.^26^ The native negative costimulatory signal naturally prevents the activation of CD8^+^ T cells to promote tolerance especially in cancers.^27^

### 2.1. Multiple Sclerosis

Multiple sclerosis (MS) is an autoimmune disease characterized by neuroinflammation, which is caused by the infiltration of autoreactive T cells into the central nervous system (CNS) that attack and damage the myelin sheath on the axons of neurons. MS patients can suffer from several forms of the disease. Relapsing-remitting MS (RRMS) is found in 87% of MS patients where patients experience periods of disease progression followed by periods of improvement.^28, 29^ RRMS patients can progress to secondary progressive MS (SPMS) without remission periods and eventually reach a point of irreversible neuronal damage with signs of axonal degeneration.^28, 29^ A small number of patients suffer from primary progressive MS (PPMS) with constant neurological deterioration and no remittance periods. Finally, progressive-relapsing MS (PRMS) is the rarest MS form with constant neurological deterioration and heightened periods of attacks.^29^

CNS infiltration by lymphocytes is one of the hallmarks of the disease in which autoreactive T cells recognize myelin sheath proteins as foreign-antigens. The myelin sheath consists of myelin oligodendrocyte glycoprotein (MOG), myelin basic protein (MBP), and proteolipid protein (PLP) that surround the axon.^6^ In the CNS, microglia cells serve as APCs for presenting self-antigens to naïve CD4^+^ T cells that differentiate to Th1 and Th17 cells.^30^ Th1 cells generate IFN-γ while Th17 cells produce IL-17 as inflammatory cytokines that stimulate immune cells to infiltrate the CNS. However, the mouse model studies indicate that IL-17-producing Th17 cells are more important than IFN-γ-producing Th1 cells in autoimmune diseases.^30^ In addition, MS patients produce a high level of IL-17 which can be used as a marker for MS.^31^

Current treatments for MS include anti-inflammatories, glatiramer acetate, and DMTs.^6^ Anti-inflammatories such as corticosteroids, IFN-β, and mitoxanthrone only help in the short-term to reduce of the inflammatory response.^6, 32^ Glatiramer acetate is an amino acid polymer that is believed to mimic MBP as a decoy that can slant the immune-response balance to a suppressor response.^33^ DMTs are normally monoclonal antibodies (mAbs) that interfere with B and T cell activity to alter the disease progression.^5^ The mAb, natalizumab, prevents CNS infiltration of immune cells by blocking interactions of α4 integrins on immune cells with vascular cell adhesion molecule-1 (VCAM-1) on the BBB endothelial cells.^5, 34^ Ocrelizumab, ofatumumab, and alemtuzumab were designed to target CD20 and CD52 and lyse immune cells.^5^ Several of these mAbs (i.e., ocrelizumab, alemtuzumab, and natalizumab) have anti-inflammatory activities that reduce the rates of relapses. While mAb treatments help suppress MS, natalizumab has a side effect of general immunosuppression and it induces progressive multifocal leukoencephalopathy (PML) in a small number of patients.^35^ This side effect can be dangerous to patients. Because of the PML side effect, there is a need to develop a specific treatment that is safer for MS patients.

### 2.2. Rheumatoid Arthritis

Rheumatoid Arthritis (RA) is another type of autoimmune disease that creates chronic inflammation of the joints due to T cell activation of self-antigen epitopes of the synovium.^36^ As in MS, proliferation of Th17 cells and upregulation of the IL-17 cytokine are related to the chronic inflammation in RA. Disease-modifying antirheumatic drugs (DMARDS) help avert the onset immune responses to counteract the disease. Abatacept is a fusion protein drug made from a conjugate between the CD80/86-Fc-binding region of IgG1 and the extracellular domain of CTLA-4. This conjugate blocks interaction of CD80/86 to CD28 as costimulatory signals and suppresses the activation of inflammatory T cells (Figure 2).^37^ Several mAbs (i.e., infliximab, etanercept, adalimumab and certolizumab) have been used as DMARDS to reduce the levels of TNF-α in RA. Anti-CD20 mAb, rituximab, binds to CD20 to reduce the B cell population for suppressing disease symptoms and promoting remission in RA.

### 2.3. Type-1 Diabetes (T1D)

In type 1 diabetes (T1D), insulin-producing β cells are attacked by autoreactive CD4^+^ and CD8^+^ T cells that infiltrate the pancreatic islets in a process called insulitis.^38^ Several self-antigens such as glutamic acid decarboxylase (GAD), insulinoma antigen-2 (IA-2), insulin, and zinc transporter 8 (ZNT8) stimulate autoreactive T cells in T1D. Suppression of GAD65 expression in beta cells could prevent diabetes in non-obese diabetic (NOD) mice.^39^ Prediabetic patients produce antibodies specific to these antigens where insulin is the primary antigen before the appearance of sensitivity to other antigens.^38^ These antigens are recognized by naïve T cells for their differentiation to inflammatory T cells (i.e., Th1 and Th17). It is interesting that soluble peptides from these antigens have been developed and successfully induced immunotolerance to T1D in animal models.

## 3. Antigen-Specific Targeting of T cells

**Figure 4.**
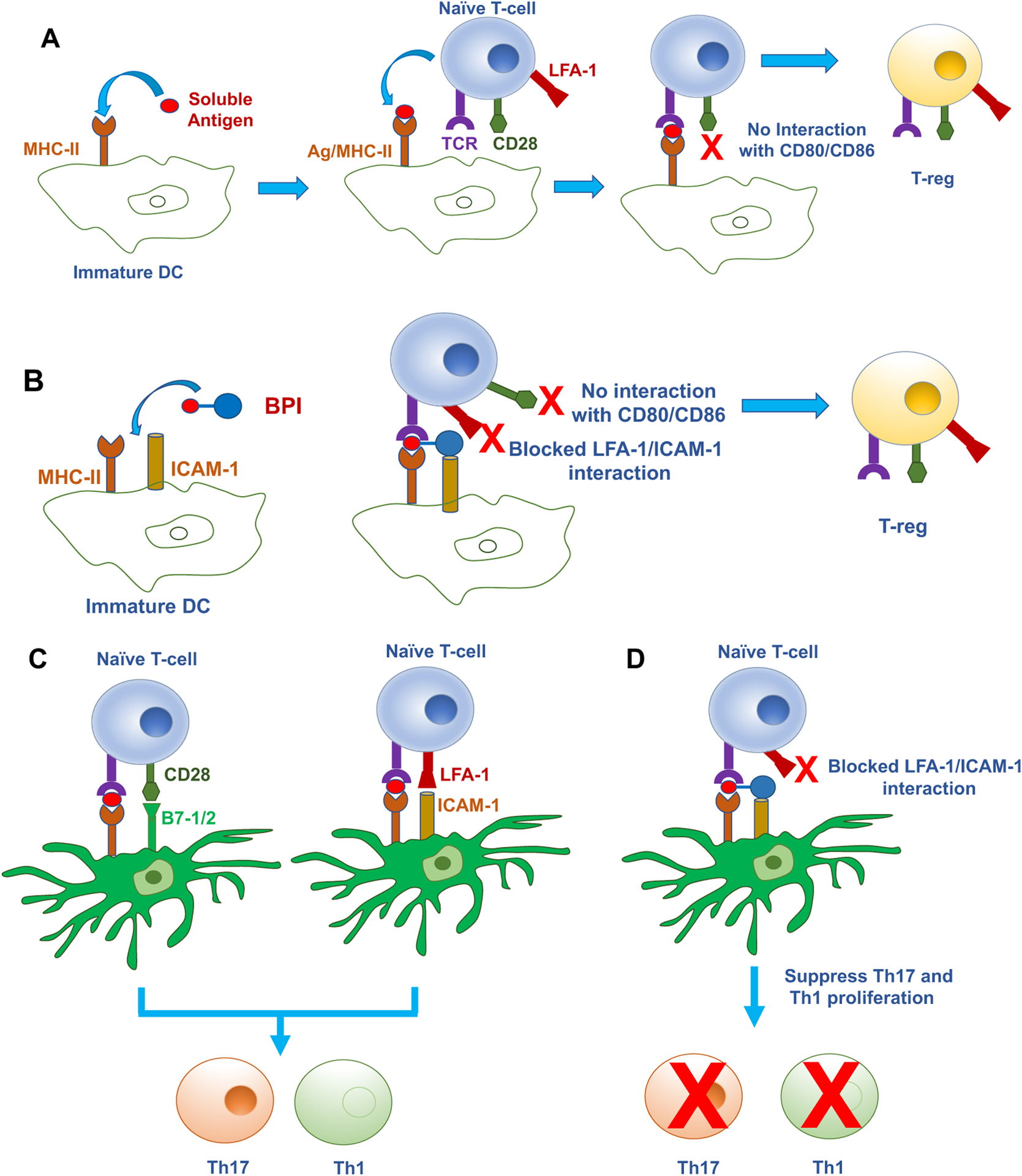
The potential mechanisms of action of antigenic peptides and BPI molecules to suppress autoimmune disease by controlling the autoreactive inflammatory T cells. **(A)** The antigenic peptide binds to empty MHC-II on the surface of immature DC (iDC). Binding of the naïve T cell to the iDC, using TCR to Ag-MHC-II in the absence of binding between CD28 to CD80/86, causes the differentiation of naïve T cells to Treg cells. **(B)** The BPI binds to both empty MHC-II and ICAM-1 on the surface of iDC. The binding of the naïve T cell to the iDC via the TCR/Ag-MHC-II complex in the absence of costimulatory signals from LFA-1/ICAM-1 and/or CD28/CD80 or CD28/CD86 results in production of Treg cells. **(C)** Binding of the naïve T cell to the mature DC (mDC) using TCR/Ag-MHC-II and costimulatory signals (Signal-2) from LFA-1/ICAM-1 and/or CD28/CD80 or CD28/CD86 produces inflammatory Th17 and Th1 cells. **(D)** The BPI binds to both empty MHC-II and ICAM-1 on the surface of the mDC. Binding of the naïve T cell to the mDC via the TCR/Ag-MHC-II complex in the absence of costimulatory signal from LFA-1/ICAM-1 results in suppression of inflammatory Th17 and Th1 proliferations.

As mentioned previously, suppression of the general immune response is a common side effect of many drugs for autoimmune diseases, and thus, patients become susceptible to opportunistic infections.^7^ One way to avoid general suppression of the immune response is by developing antigen-specific treatments characteristic to a specific autoimmune disease. Because autoreactive proinflammatory T cells are initiated from the activation of naïve T cells, the goal is to selectively suppress the autoreactive T cells in an antigen-specific manner while stimulating the proliferation of regulatory cells (i.e., Treg). During naïve T cell–APC interaction, T cell activation begins after the IS formation (Figure 3); dendritic cells (DCs) normally have an important role in presenting the processed peptide antigens (Figure 4). However, it has been shown that delivering soluble peptide epitopes of an antigenic protein can suppress a subpopulation(s) of reactive immune cells (i.e., T cells and B cells) that recognize the antigen (Figure 4A).

### 3.1. Costimulatory Signals in Autoimmune Diseases

The important role of the costimulatory signal (Signal-2) has been elucidated by showing the suppression of inflammatory immune cell activation by blocking this signal in animal models of autoimmune diseases. Inhibition of ICAM-1/LFA-1 interactions (Signal-2) by antibodies, peptides, and small molecules modulates T-cell activation to suppress T1D,^40, 41^ psoriasis,^42, 43^ RA,^44, 45^ and allograft rejection^46, 47^ in animal models (Figure 3D). Administration of anti-ICAM-1 and anti-LFA-1 mAbs together inhibits allograph rejection of heart transplantation between two different strains of mice.^46^ The results indicate that blocking the ICAM-1/LFA-1 signal (Signal-2) interaction prevents activation of inflammatory T cells that attack the foreign organ from a different strain of mice (Figure 3D).^46^ In this case, blocking Signal-2 in the presence of Signal-1 induces T cell anergy by preventing the complete formation of IS (Figure 3D). It has been shown that psoriasis can be treated with an anti-CD11a mAb (efalizumab) that blocks the ICAM-1/LFA-1 signal (Signal-2).^48–50^ Unfortunately, the side effect of efalizumab was progressive multifocal leukoencephalopathy (PML), which is often fatal, in a population of patients; in 2009, the use of this drug was terminated.^51, 52^

Natalizumab (Tysabri®) was developed for treatment of MS. This mAb binds the α4-subunit and blocks the α4β1 and α4β7 integrins interactions on leukocytes to VCAM-1 molecules of the vascular endothelial cells of the blood-brain barrier (BBB) to inhibit leukocyte adhesion to the BBB endothelial cells.^53, 54^ As a result, it prevents the infiltration of immune cells into the brain and halts disease progression in MS. The use of natalizumab was halted for a short period of time because it caused a small population of MS patients to develop PML. It was speculated that blocking integrin activity could lower the activity of the general immune response to fight pathogenic infections. Therefore, an alternative solution in treating MS patients without compromising their general immune systems will benefit patients. In this case, a new and novel method is needed to selectively suppress only a subpopulation of autoreactive T cells that react to a specific self-antigen in a specific autoimmune disease. As a result, such treatments will avoid suppression of the general immune responses.

As mentioned previously, positive and negative costimulatory signals alter T cell activation.^13, 15, 19, 55^ Interactions between CD28 and CD80/86 are one of the most important positive costimulatory signal that leads to sustain activation of proinflammatory T cells.^15, 55–57^ It was proposed that blocking this signal could be a method for specific immunomodulation. Alternatively, CD80/86 can also interact with CTLA-4 as a negative costimulatory signal and this signal has been shown to suppress the activation of inflammatory T cells in cancers. A fusion between CTLA-4 and the Fc region of IgG1 has been shown to induce T cell anergy *in vitro*; however, it did not modulate T cell activation *in vivo*.^15^

### 3.2. Antigenic Peptides for Autoimmune Diseases

#### 3.2.1. Natural Antigenic Peptides:

Administrations of soluble antigenic peptides in animal models have been shown to suppress various autoimmune diseases; they act in an antigen-specific manner and avoid suppression of the general immune response. The antigenic peptides mimic T cell epitopes with linear sequences (<20 amino acids) that induce tolerogenic effects via immune deviation.^58–60^ Their small size allows them to evade immunogenicity and they are not able to cross-link for IgE activation.^60^ It is critical to note that the antigenic peptide must be soluble because insoluble peptide could induce local inflammation.^60^ For example, the GAD_208–217_ peptide (Table 1) from GAD65 epitope suppressed T1D in NOD mice and the peptide complexed with empty I-A^g7^ MHC-II and HLA-DQ8 MHC-II on the surface of APC.^61, 62^ The soluble antigenic peptides prevent inflammatory T cell activation and insulitis to protect insulin-producing β cells in T1D animal models. Similarly, antigenic peptides from myelin sheath proteins can suppress the progression of multiple sclerosis in animal models. For example, PLP_139–151_ peptide from an epitope of myelin sheath PLP suppresses experimental autoimmune encephalomyelitis (EAE) disease symptoms in mice as an animal model for MS.

Designing antigenic peptides for immunotolerance is more complex than administering a fragment of the protein antigen.^63^ Antigenic peptides can be administered to stimulate upregulation of different T cell phenotypes without needing the whole protein antigen. These antigenic peptides can induce tolerance towards the antigen by increasing the number of Tregs rather than inflammatory T cells. Antigenic peptides from epitopes of the antigen protein with 13–17 amino acids have the best fit to MHC-II binding pocket. At times, a newly designed peptide from the antigenic protein may not have immunological activities as the naturally processed antigenic peptides; this is because the newly designed peptide has a different conformation than the peptide epitope on an intact protein antigen.^64^ Thus, the conformation of antigenic peptide (apitope) must match that of the naturally processed epitope that stimulates T cells without antigen processing.^63^

Different antigenic peptides in MS were thought to be linked to different forms of MS.^65^ Different types of EAE as different models of MS can be stimulated by PLP, MBP, or MOG peptide in complete Freund’s adjuvant (CFA). Activations of PLP- and MOG-specific autoreactive T cells have been proposed to be correlated with RRMS and SPMS, respectively. In MS, MBP-specific autoreactive T cells are also stimulated as a result of epitope spreading.^65^ Administration of PLP peptides suppressed in animal models for RRMS while MOG peptides suppressed animal models for SPMS. Thus, different antigenic peptides could be targeted to various stages of MS. Due to antigen specificity, the MOG peptide can only suppress MOG-stimulated EAE, not PLP-stimulated EAE or MBP-stimulated EAE.^66^ Due to the antigenic specificity of this approach, it may be necessary to administer a mixture of multiple antigenic peptides for a more robust regulatory response to suppress MS.^58^

The mechanism of antigenic peptide is due to its binding to empty unprocessed MHC-II on the surface of immature dendritic cells (iDCs) to form peptide/MHC-II complex (Figure 4A).^59, 60^ TCRs on naïve T cells then bind to the peptide/MHC-II complexes on the iDCs in the absence of costimulatory molecules (i.e., CD80/CD86). The peptides bind to empty MHCs without regular processing through the cytoplasmic domain; therefore, the naïve T cells become regulatory T cells (Treg) that produce regulatory cytokines (i.e., IL-10, IL-35, TGF-β) to provide a tolerogenic response (Figure 4A). The upregulation of Tregs produces regulatory cytokines that suppress the activation of Th1 and Th17 cells in an antigenic-specific manner.^58^ In autoimmune diseases, the disease onset is due to the skewed balance of inflammatory (i.e., Th1, Th17) over regulatory (i.e., Treg) immune cells. Therefore, an important feature when trying to suppress autoimmune diseases is to stimulate the proliferation of Tregs. Yu *et al*. showed that suppression of autoimmune diseases cannot be done without the presence of Tregs.^67^ Furthermore, the upregulation of Tregs was able to suppress EAE in an antigen-restricted manner.^67^ Treg cell proliferation has been associated with the upregulation of regulatory cytokines such as IL-10, IL-35, and TGF-β as well as FoxP3 and CD25 markers on T cells.^68, 69^ IL-10 reduces the BBB’s disruption and leakiness in MS and decreases CNS infiltration of proinflammatory molecules and immune cells through the BBB. Thus, the administration of antigenic peptides leads to a specific immunotolerance without suppressing the overall immune system.^63^

The EAE disease has been developed in rats and mice as animal models for MS to evaluate potential therapeutics for MS. These animal models have been used to find different active peptide epitopes of the PLP antigen protein, including PLP_130–147_, PLP_139–154_, PLP_137–151_, PLP_139–151_ and PLP_103–116_. Immunization of animals with the different PLP peptides in CFA can induce EAE.^70^ The nasal administrations of soluble MBP peptide in rats suppressed EAE.^58^ Peptide epitopes from MBP and MOG were also found to be active. These antigenic peptides could be delivered via intravenous (i.v.), subcutaneous (s.c.) or oral route to suppress EAE. For the oral route, the antigenic peptides need to be administered as a vaccine, indicating that vaccine delivery of peptide is necessary to prime naïve T cells to become regulatory T cells (Treg) for suppressing the disease. A co-administration of four different MBP peptides as ATX-MS1467 (MBP_30–44_, MBP_30–44_, MBP_131–145_, and MBP_140–154_) had reached clinical for SPMS; during a phase-I study, patients treated with these peptides showed improvement from the disease symptoms.^71, 72^

Soluble antigenic peptides derived from collagen II (CII) such as CII_256–270_, CII_707–721_, and CII_1237–1249_ can suppress RA in animal models by suppressing inflammatory T cell activity.^36^ Collagen II is found in the synovial joints and can elicit an inflammatory T cell response in RA.^73–75^ Both CII_256–270_ and CII_707–721_ suppressed RA scores compared to the control in the CIA model while the CII_1237–1249_ peptide did not suppress the disease symptoms.^36^ The activities of CII_256–270_ and CII_707–721_ were presumably due to their binding to I-A^g7^ MHC-II molecules on APCs (i.e., dendritic cells).^75–77^ Although molecular modeling with the X-ray structure of DR4 MHC-II has shown that the CII_1237–1249_ peptide can form a peptide MHC-II complex, it does not have activity *in vivo*.^78^ Therefore, peptide binding to MHC-II doesn’t necessarily modulate the immune response.^78^ CII_256–270_ lowered the IL-6 levels in the serum indicating suppression of Th17 cells.^36^ It should be noted that Th17 cells are also important in the inflammatory response in CIA.^79, 80^

#### 3.2.2. Altered Peptide Ligands (APLs):

Altered peptide ligands have been developed as an alternative to soluble natural antigenic peptides to improve regulatory immune responses compared to natural antigenic peptides. The initial idea behind dosing APLs is that they can interrupt the interaction between the TCR and MHC-II to prevent the progression of EAE and other autoimmune diseases.^81^ Altering the sequence of the native antigenic peptide to make the APL can potentially modulate TCR/Ag-MHC-II interactions with the hope that the APL is more effective than the native antigenic peptide.^81^ In APLs, the amino acids of the parent peptide are mutated to improve binding to MHC-II and/or enhance the binding of Ag-MHC-II complex to TCR.^82^ The best known APL is the glatiramer acetate polymer, which is currently marketed as Copaxone® for treating MS.^83^ The polymer was designed to mimic the MBP antigenic peptide with randomly polymerized L-alanine, L-glutamic acid, L-lysine, and L-tyrosine.^83, 84^ The potential side effect of repeated administrations of APL such as glatiramer acetate is that a small population of patients developed hypersensitivity reactions, specifically anaphylaxis.^85^

Naturally occurring APLs, that are generated by a single amino acid modification via a naturally occurring reaction(s), can also be responsible for the onset of autoimmune diseases.^86^ This natural APL causes altered interactions between TCR and Ag-MHC-II to form TCR/Ag-MHC-II complexes.^86^ These modifications that can form APLs include citrullination, deamidation, and deimidation.^86–89^ The citrullination reaction specifically changes the charge of the overall peptide due to the conversion of the positively charged arginine residue to a neutral citrulline residue; this alters the APL binding properties to MHC.^86^ Citrullination is caused by peptidyl arginine deiminases (PAD), which are normally overexpressed during inflammation by inflammatory cells.^86, 90^ Post-translational modifications have been associated with an increase in peptide binding affinity to MHC-II as well as the onset of autoimmune diseases (i.e., RA and MS).^86, 91, 92^ Deamidation of gliadin peptides by transglutaminase 2 (TG2) enzyme could alter the peptide conformation and increase peptide affinity to MHC-II that causes the induction of a Th1 response, known as Celiac disease.^86, 93–96^

It was previously believed that blocking MHC-II with an APL was enough to prevent autoimmune disease; however, blocking of MHC alone is nonspecific and could lead to suppression of other immune responses (i.e., innate and humoral responses).^81, 97^ Because TCR genes are highly conserved, even between different rodent species, TCRs could be a better target for more specific autoimmune therapies.^98–100^ In addition, another important characteristic of T cells is that they can recognize multiple ligands. Thus, it allows soluble antigenic peptides to be mutated so that they can still be recognized by the TCR while inducing a different immune response. Therefore, generating TCR antagonists using APLs can be developed for preventing inflammatory T cell activation.^81^ Antagonist peptide sequences are determined by replacing a single amino acid on the antigenic peptide to see which peptide still binds to the TCR.

Substitutions of a single amino acid residue in the PLP_139–151_ peptide (Table 1) to make APLs allowed for the discovery of critical residues for binding to MHC-II and TCR using MHC and TCR binding assays, respectively.^81^ Because of their potential to modulate immune responses, APLs from the PLP peptide were developed as potential therapeutics to treat MS.^101^ Residues W144 and H147 in PLP_139–151_ were respectively replaced with L144 and R147 to make the LR-APL (Table 1) with increased TCR binding properties.^81^ The efficacy of LR-APL was compared with the parent PLP_139–151_ in suppressing EAE in mice; the result showed T cells activated by LR-APL accessed the CNS and had different antigen recognition than those of activated by the native PLP_139–151_ peptide.^102^ It was previously suggested that T cells activated by LR-APL cross-reacted with TCRs specific for the native PLP_139–151_ peptide.^102^ In the EAE model, a lower number of PLP-specific T cells were found in the CNS of mice that were co-immunized with both PLP_139–151_ peptide and LR-APL peptide compared to those that were only immunized with the PLP_139–151_ peptide alone.^102^ The result suggests that LR-PLP was able to inhibit the PLP-specific T cells from activation so that less PLP-reactive T cells were activated and infiltrated the CNS. The success of LR-APL was attributed to its activation of cross reactive T cells in the CNS to allow for the suppression of the symptoms of EAE. Unfortunately, LR-APL did not increase the regulatory cytokines in the CNS and it was proposed that further study was necessary to determine the upregulation of regulatory cytokines in the periphery to elucidate the LR-APL mechanism of action. Later, LR-PLP was compared to the parent PLP_139–151_ peptide and PLP-BPI in the PLP-stimulated EAE mouse model for RRMS.^103^ It was found that LR-PLP exacerbated the disease in EAE worse than PBS, while PLP_139–151_ peptide suppressed the disease symptoms significantly better than the PBS-treated mice.^103^ At the same dose, the PLP-BPI suppressed the disease symptoms significantly better than those of PLP_139–151_, LR-PLP, and PBS.

Similarly, APLs from the allergen Par J1 were developed by substituting its residues with alanine and valine amino acids to alter binding to MHC-II, and also interfere with TCR interactions.^104^ Thus, the APLs behaved as TCR antagonists rather than solely MHC antagonists. APLs specific to TCR do not all work the same way because they can be antagonists, partial agonists, or super agonists depending on the sequence.^105^ Although APLs as partial agonists cannot induce T cell proliferation, they can induce cytokine release and generate a Th2 response rather than a Th1 response.^86, 106^ APLs as antagonists decrease T cell proliferation by acting as competitive inhibitors to induce a negative feedback loop.^105, 107, 108^ APL antagonists were selected because of their binding properties to MHC-II while inhibiting activation of inflammatory T cells. Some of the antagonist APLs inhibited the proliferation of T cells to stop the induction of EAE in animal models.^81, 82^ Administration of APLs also led to the upregulation of Treg cells reflected by production of IL-10.^109, 110^

In a short clinical trial, MBP_83–99_ and an APL derivative called NBI-5788 were evaluated to understand their effects on immediate as well as long-term immune deviation. The treatments reduced the amount of lesions in the brain and shifted the immune response from Th1 to Th2 in a group of MS patients.^111, 112^ MS patients who received placebo showed low levels of APL-reactive T cell with high levels of the inflammatory cytokine, IFN-γ.^112^ These same observations were made for MS patients that received MBP_83–99_. For the 5 patients who received NBI-5788, an increase in T cell immune response to the APL was observed both in the short-term and long-term. Two of these patients, with strong and elevated immune responses to the APL, showed high levels of cross-reactivity between the APL, MBP_83–99_ peptide, and MBP protein. The high levels of cross-reactivity showed the potential to induce tolerance to the native protein in the long-term. APL-reactive cells produced more IFN-γ while the MBP-reactive cells produced a mix of IFN-γ and IL-5.^112^ In addition, patients that had little to no response from the APL in the short-term did not eventually develop an APL response in the long-term. Unfortunately, APLs have the side effects of anaphylaxis or hyperreactivity reactions in treated MS patients.^86, 113^ Other side effects in a clinical trial of NBI-5788 were that 10% of patients developed rash, abdominal pain and nausea. Another clinical trial of MBP APL (CGP77116) did not improve prognosis in MS patients. This molecule increased Th1 response, which presumably due to the high administered dose of APL that caused a strong APL/TCR interaction.^114–117^

In a similar study, the activity of MBP_87–99_ peptide was compared to four APL analogs including the [R^91^, A^96^]-MBP_87–99_ and [A^91^, A^96^]-MBP_87–99_ peptides and their mannan conjugates in the EAE mouse model.^118^ MBP_87–99_-treated mice increased the level of IFN-γ while treatment with the [R^91^, A^96^]MBP_87–99_ mannan conjugate reduced the levels of IFN-γ.^118^ In addition, MBP_87–99_ did not induce the upregulation of regulatory cytokine IL-4; in contrast, the levels of IL-4 were increased upon treatments with APL analogs. The EAE mice treated with the mannan-[R^91^, A^96^]-MBP_87–99_ conjugate produced higher levels of IL-4 than those of treated with just [R^91^, A^96^]-MBP_87–99_.^118^ Although mice treated with the other analog (i.e., [A^91^, A^96^]-MBP_87–99_) produced higher levels of IL-4 compared to those treated with MBP_87–99_, the [A^91^, A^96^]-MBP_87–99_-treated mice still had high levels of IFN-γ or similar to those treated with MBP_87–99_.^118^ However, the mice treated with mannan-[A^91^, A^96^]-MBP_87–99_ conjugate had both increased IL-4 levels and decreased IFN-γ levels. Thus, it was found that the mannan conjugates shifted the immune response from Th1 to Th2. Further study is still needed to explain why the mannan-[A^91^, A^96^]-MBP_87–99_ conjugate diverted the immune response from Th1 to Th2. It was also found that the conjugate reduced pro-inflammatory cytokine IL-17 due to upregulation of Treg cells.^118^

Cyclo-(87–99) [A^91^, A^96^]-MBP_87–99_, which is a cyclic analog of [A^91^, A^96^]-MBP_87–99_, was evaluated in the EAE model in male and female rats. The treated male and female rats with the cyclic analog had lower clinical scores that those treated with MBP_87–99_.^119^ The EAE clinical scores of female rats treated with MBP_87–99_ were higher than male rats.^119^ Animals treated with cyclo-(87–99) [A^91^, A^96^]-MBP_87–99_ and [A^91^, A^96^]-MBP_87–99_) had a higher pain threshold compared to those treated with the native MBP_87–99_ peptide. The increase in pain threshold from the APLs was attributed to the reduced levels of the proinflammatory cytokines IFN-γ and TNF-α as well as the increased levels of regulatory cytokines IL-4, IL-10, and IL-13.^119^

Several mechanisms have been proposed on how APLs work to antagonize the TCR. One hypothesis was that the antagonist activity of the APL involves only activating the CD3 costimulatory complex without activating multiple costimulatory complexes.^120^ The second hypothesis indicates that APLs reduce the tyrosine phosphorylation of the TCR ζ-chain and alters the activation of T cells.^121^ APLs can also influence the expression of CD40L on activated T cells.^122^ It has been shown that some APL antagonists increased mononuclear cells in the CNS, indicating that this approach could be immunogenic and did not serve the intended purpose of the APL. The third hypothesis is that the conformation of the APLs can trigger the activation of type-B T cells in the periphery without antigen processing by APC, leading to the onset of autoimmune diseases.^64, 86, 123^ Because APLs, while binding to MHC-II, can have a different hydrogen bonding scheme than those of parent antigenic peptides, these resulting structural differences increase the number of MHC complexes on the surface of the APC.^118, 124^ As a result, the increase in APL loading on MHC-II can interfere with both TCR binding and T cell stimulation.

## 4. Bifunctional Peptide Inhibitors

As mentioned previously, the costimulatory signal (Signal-2) has been modulated to skew T cell commitment between inflammatory and regulatory phenotypes.^125^ Blocking the LFA-1/ICAM-1 signal using antibodies suppresses autoimmune diseases such as type-1 diabetes,^41^ rheumatoid arthritis,^44, 45^ and psoriasis.^43^ This also opens opportunities to develop peptides, peptidomimetics, and small molecules to block LFA-1/ICAM-1 signal.^125–130^ Peptides derived from the domain 1 (D1) of ICAM-1 can block LFA-1/ICAM-1-mediated T cell adhesion as well as mixed lymphocyte reaction by binding to LFA-1.^130–133^ Similarly, LFA-1 peptides derived from CD11a (α-subunit) or CD18 (β-subunit) can inhibit LFA-1/ICAM-1-mediated T cell adhesion and mixed lymphocyte reaction.^134–139^ Unfortunately, blocking a costimulatory signal such as LFA-1/ICAM-1 interactions could suppress the general immune response. Therefore, targeting a specific subpopulation of T cells that are activated in a specific autoimmune disease may be necessary.

**Figure 5.**
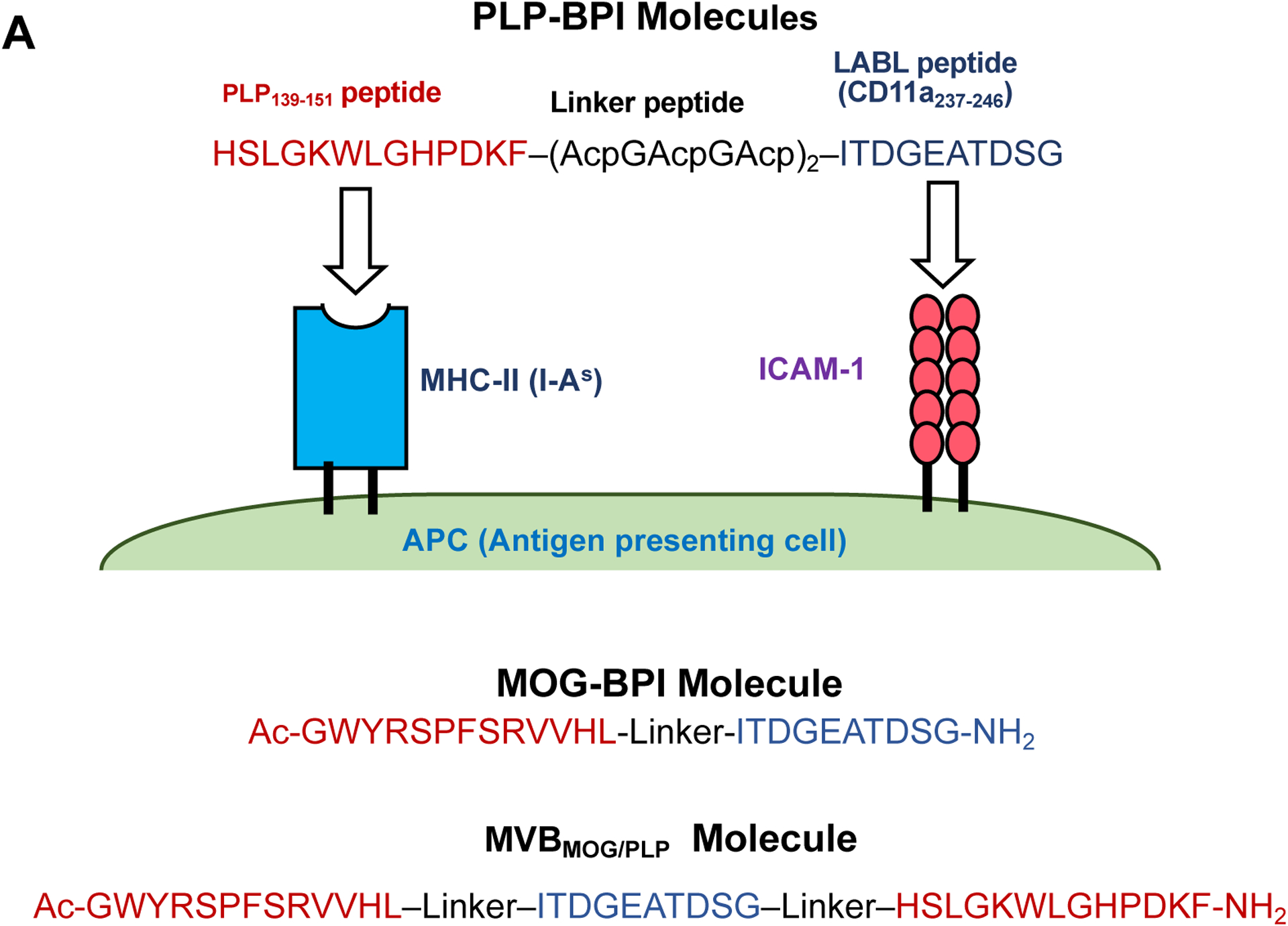

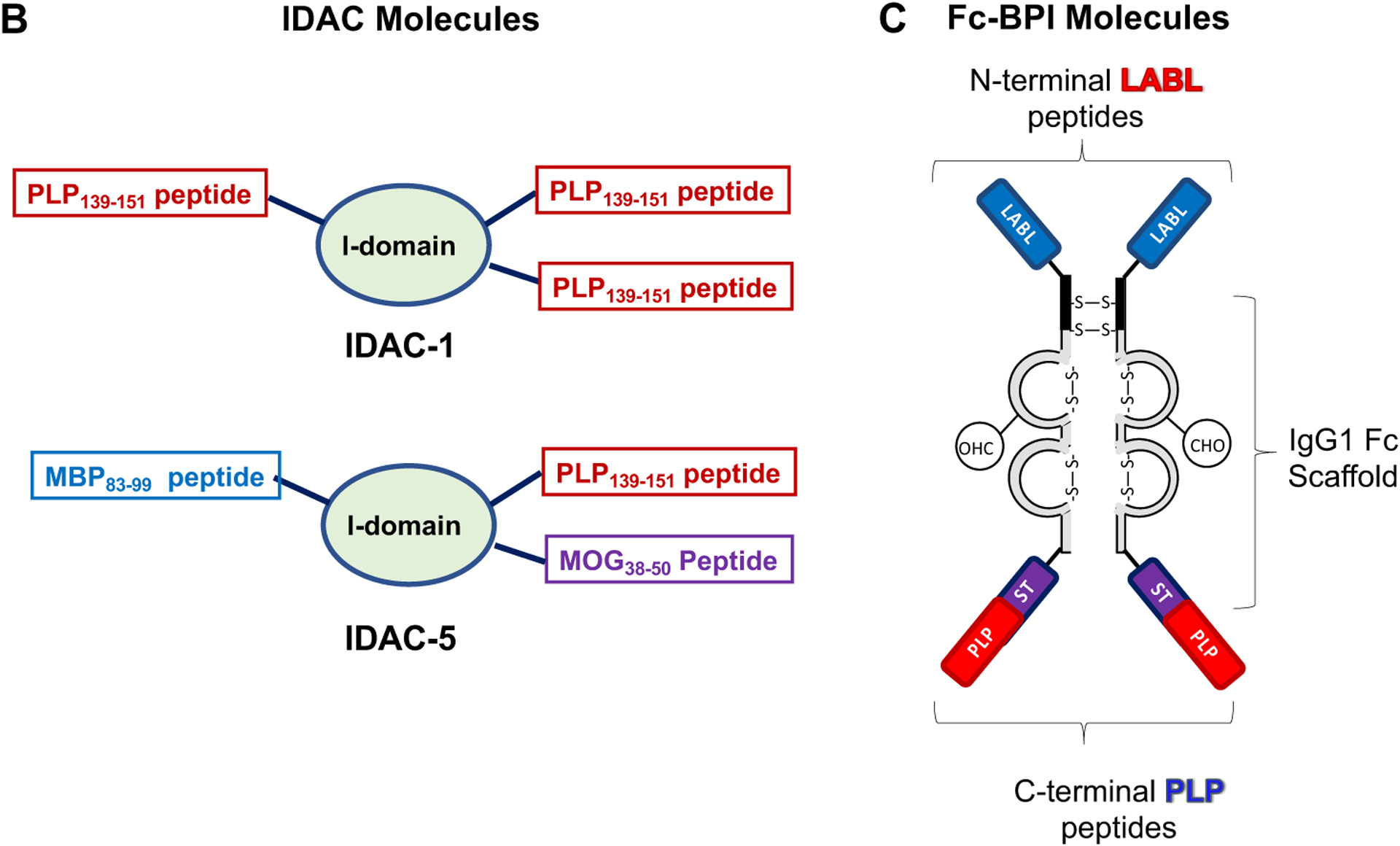
BPI molecules consists of an antigenic peptide(s) conjugated to LABL peptide or I-domain protein. **(A)** PLP-BPI is a conjugate between PLP_139–151_ and LABL peptides. The antigenic peptide binds to MHC-II while LABL peptide (CD11a_237–246_) binds to ICAM-1 domain 1 (D1). This simultaneous binding prevents immunological synapse (IS) formation. MOG-BPI is a conjugate between MOG_38–50_ and LABL peptides. MVB_MOG/PLP_ is a BPI molecule containing two antigenic peptides and LABL peptide. **(B)** The structure of another form of BPI called I-domain antigen conjugates (IDAC), in which antigenic peptides are conjugated to lysine residues of the I-domain. IDAC-1 is a conjugate between I-domain and PLP_139–151_ peptides. IDAC-5 is a conjugate between I-domain and multi-antigenic peptides from such as PLP_139–151_, MOG_38–50_ and MBP_83–99_. **(C)** The Fc-BPI structure: the Fc domain contains two LABL peptides at the N-terminus and two antigenic peptides PLP_139–151_ at the C-terminus.

To achieve the targeting of a sub-population of immune cells, various types of bifunctional peptide inhibitors (BPIs) were designed by conjugating an antigenic peptide and a Signal-2 peptide or protein (i.e., the LABL peptide and I-domain of LFA-1)(Figure 5). The idea is that a BPI will bind to the surface of an APC by interacting with empty MHC-II via the antigenic peptide and simultaneously bind to ICAM-1 via the LFA-1 peptide known as LABL, derived from CD11a (237–247) (Figure 5A). In BPIs, antigenic peptides are separated from the LABL peptide via a long linker using several aminocaproic acid or polyethylene glycol based linker (Figure 5A). The required linker length for simultaneous binding was estimated from molecular modeling using X-ray structures by docking of the antigenic peptide to MHC-II and LABL peptide to the D1 domain of ICAM-1 where these two proteins were both anchored to cell membranes. Thus, the BPI will disrupt the cluster formation of pSMAC as well as cSMAC to inhibit the IS formation (Figure 3E). The inhibition of the IS formation suppresses the proliferation of Th17 and Th1 cells while stimulating antigen-specific Treg upregulation (Figures 4B–C). The presence of the antigenic peptide on BPIs ensures they selectively target a subpopulation of disease specific T cells as well as avoiding suppression of general immune responses to other antigens.

### 4.1. GAD-BPI for T1D

The concept of the BPI was tested using the GAD-BPI molecule by conjugating the GAD65_208–217_ peptide with the LABL peptide (CD11a_237–246_) via a linker that is made from a combination of aminocaproic acid and glycine (Table 1).^140^ The GAD_208–217_ peptide binds to I-A^g7^ MHC-II on APCs. On the surface of APC, GAD-BPI molecules block the IS formation while GAD peptide/MHC-II complexes are still recognized by TCRs on a specific population of naïve T cells. Therefore, other subpopulations of T cells with TCRs that recognize other antigens (i.e., pathogens) will not be suppressed and can still be activated to fight an infecting pathogens. As a result, GAD-BPI and other BPI molecules do not suppress general immune responses. To test the activity of GAD-BPI, a T1D mouse model was developed by administration of the GAD_208–217_ peptide (40 nmol) in complete Freund’s adjuvant (CFA) via subcutaneous (s.c.) route. Administration of GAD-BPI (80 nmol, 100 μL) on days 0 and 7 significantly suppressed insulitis in T1D mice compared to control mice injected with phosphate buffer saline (PBS).

GAD-BPI-treated mice had low insulitis with 83% of normal islets on day 8 while PBS-treated mice had high insulitis with only 35% of normal islets.^39, 140^ Next, CD4^+^ T cells isolated from GAD-BPI-treated and PBS-treated NOD mice were transferred (adoptive transfer) to evaluate their effects in NOD severe combined immunodeficiency (NOD.Scid) mice. About 72% NOD.Scid mice that were received CD4^+^ T cells from GAD-BPI-treated mice did not have hyperglycemia (*i.e*., blood glucose ≥ 250 mg/dl) after 7 weeks while only 17% of NOD.Scid mice did not have hyperglycemia when treated with CD4^+^ T cells from PBS-treated mice. The mice receiving CD4^+^ T cells from GAD-BPI-treated mice had significantly lower insulitis compared to those that received CD4^+^ T cells from PBS-treated mice.^140^ Incubation of isolated B cells from NOD mice with GAD-BPI had high spots of colocalization between I-A^g7^ MHC-II and ICAM-1 when evaluated by confocal microscopy studies.^140^ This suggests that the GAD-BPI can simultaneously bind to MHC-II and ICAM-1 on the surface of B cells or APCs and can disrupt the IS formation.

### 4.2. PLP-BPI and MOG-BPI in EAE mouse models

#### 4.2.1. Comparison of the In Vivo Activities of PLP-BPI, PLP, LR-APL, LABL, and PBS:

To test the general application of the BPI concept for other autoimmune diseases, the activity of a PLP-BPI (Table 1, Figure 5A) in controlling disease progression in the EAE mouse model was evaluated. As a model for RRMS, SJL/J mice were immunized to stimulate EAE disease using PLP_139–151_ in CFA.^103^ The disease symptom normally starts on day 9 after immunization with the peak of disease exacerbation between days 13 and 15; then, followed by disease remission around day 20 and relapses starting on day 40. After disease stimulation, PLP-BPI (100 nmol/injection) was injected via the tail vein on days 2, 7, 10, and 14 and it was compared to PLP_139–151_, PLP_L144, R147_ (or LR-APL), OVA-BPI, and LABL peptides at the same dose (Table 1). Besides the disease scores, the activity of the BPI was observed using the body weight changes. PLP-BPI-treated EAE mice exhibited very low disease scores or lower than those treated with the PLP_139–151_ peptide.^103^ High disease scores on the peak of disease were observed in mice treated with PLP_L,R_ (LR-APL), OVA-BPI, and LABL peptides. Co-administrations of the PLP_139–151_ and LABL peptides suppressed the disease better than the PLP_139–151_ peptide alone, indicating a synergistic effect of both peptides. The OVA-BPI peptide with unrelated antigenic peptide did not suppress the disease, proving the need of an appropriate antigenic peptide for the suppressive activity.^103^ PLP_L,R_, intended as TCR antagonist and a positive control, did not suppress the EAE.^81^ PLP-BPI-treated mice had body weight loss of 10% compared to 24% in the PBS control on days 13–15 (disease peak). The PLP-BPI-treated mice had a lower disease incidence compared to the control groups (i.e., PLP_L,R_, OVA-BPI and PBS).

Because autoimmune disease patients will normally show signs of disease before receiving treatments, BPI administrations for three consecutive days were delivered after the observed disease symptoms began. Thus, the animals were injected with BPI when initial disease symptoms were observed and stopped when remission was started.^141^ Treatments with three BPIs (i.e., Ac-PLP-BPI-PEG3, Ac-PLP-BPI-PEG6, Ac-PLP-BPI-NH_2_-2; Table 1) for three consecutive days showed quick recovery from the disease before reaching the peak of the disease and before the normal remission time on day 20.^141^ Recovery was observed immediately after the first injection of all three BPIs, and when the 3 injections were completed, the mice had very low disease scores or were close to that of heathy animals within 2–3 days.^141^ As expected, PBS-treated mice had disease exacerbation without recovery from the disease peak until the normal remission days. Overall, BPI treatments reversed disease exacerbation when treated after the observed disease symptoms began.

#### 4.2.2. BPI Selectivity and Multi-antigen BPI:

BPI antigenic selectivity was tested using PLP- and MOG-stimulated EAE mouse models for RRMS and SPMS, respectively. MOG-BPI (Table 1) suppressed the disease in MOG-stimulated EAE but did not suppress the disease in PLP-stimulated EAE.^65^ Similarly, Ac-PLP-BPI-NH_2_-2 effectively suppressed the disease exacerbation of PLP-stimulated EAE but did not effectively suppress MOG-stimulated EAE.^65^ To design a multiantigen peptide, the LABL peptide was simultaneously conjugated to both the PLP_139–151_ and MOG_38–50_ peptides via polyethylene glycol (PEG)-glycine linkers to make a multivalent BPI (MVB_MOG/PLP_) molecule (Figure 5A, Table 1).^65^ The multiantigen BPI, MVB_MOG/PLP_, was active in suppressing both PLP- and MOG-stimulated EAE, demonstrating that MVB_MOG/PLP_ simultaneously delivers two different antigenic peptides to alter immune cells in both the RRMS and SPMS animal models. The efficacy of MVB_MOG/PLP_ was better compared to MOG-BPI in the MOG-stimulated EAE, signifying that the PLP peptide in MVB has an additive effect in the SPMS mouse model.^65^

#### 4.2.3. Controlled-Release Formulation using Microparticles:

Because of the need for repeated i.v. administrations and the potential rapid clearance of Ac-PLP-BPI-NH_2_-2 or other BPIs (Table 1), alternative methods for delivery of BPI molecules were investigated. In this case, Ac-PLP-BPI-NH_2_-2 was loaded into PLGA microparticles in different doses and they were administered via s.c. route for a controlled-release formulation. The BPI particles preparation and characterization are described in Zhao *et al*.^142^ The activity of the BPI (or Ac-PLP-BPI-NH_2_-2) in solution and in loaded microparticles were evaluated in six groups of EAE mice (ten/group).^142^ As a control (Group 1), PBS-treated EAE mice had high disease scores and 20% decrease in body weight on days 10–12. The second group was injected with 4 × 100 nmol of Ac-PLP-BPI-NH_2_-2 (BPI) in solution via an i.v. route on days 4, 7, 10, and 14, respectively (Group 2). This treatment suppressed the EAE symptoms very effectively with no clinical signs of disease and no appreciable weight loss. Group 3 was injected with 4 × 100 nmol of BPI via s.c. route on days 4, 7, 10, and 14 and the treatments resulted in no symptoms and weight loss. In Group 4, the animals were injected four times (i.e., days 4, 7, 10, and 14) with 100 nmol of BPI in solution + blank particles using the s.c. route. The animals in Group 4 had similar profiles as in Groups 2 and 3. The results from Group 4 suggested that blank particles has no activity. The Group 5 mice were injected with one injection of solution BPI molecule (100 nmol) on day 4 followed by one injection of nanoparticles containing 300 nmol of BPI on day 7. Clinical scores and body weight of Group 5 mice were clearly better than untreated controls but slightly worse than those receiving Ac-PLP-BPI-NH_2_-2 in solution. Mice in Group 6 were injected on day 4 with nanoparticles loaded with 400 nmol of BPI via s.c. route and this one treatment suppressed the disease completely, resulting in healthy animals (clinical score = 0). In summary, BPI can be delivered via a single s.c. injection using a controlled-release method and this method is more convenient than multiple injections of soluble BPI via i.v. or s.c. route.

Another controlled-release method to deliver Ac-PLP-BPI-NH_2_-2 was developed using two types of nanoparticles made from a mixture of poly(D,L-lactic-co-glycolic acid) (PLGA) coated with alginate and chitosan to form colloidal gel systems. The BPI was loaded by physical mixing into the gel.^143^ The colloidal gel containing BPI was administered 11, 8, and 5 days before induction of EAE on day 0 to test the BPI efficacy as a vaccine for EAE.^143^ Compared to the PBS control and blank colloidal gel, the BPI-colloidal gel showed a high effectiveness in suppressing EAE. The disease suppression levels were further evaluated by observing the level of inflammatory and regulatory cytokines after the treatment period. The mice in the BPI-colloidal gel group had significantly lower levels of inflammatory cytokines (i.e., IL-6, IL-17, and IFN-γ) compared to the control.^143^ This was further confirmed by the increased levels of the regulatory cytokine IL-2. These results indicate that the colloidal gel method is an efficient method to deliver BPI molecules as a vaccine-like treatment as well as prophylactic or regular controlled-release treatment.

#### 4.2.4. The Effects of BPI on the BBB Integrity and Production of Cytokines in EAE Mice:

The pathologies of the CNS in EAE mice are analogous to those of MS patients in terms of the immune cells (i.e., T cells, macrophages, monocytes) present in the brain lesions because of the leakiness in the blood-brain barrier (BBB) in both diseases.^21, 144^ The BBB leakiness can be detected by magnetic resonance imaging (MRI) in which a high brain deposition of gadolinium diethylene-triamine-pentaacetate (Gd-DTPA) is observed after its i.v. delivery.^145^ The effects of the BPI in preventing the BBB leakiness was also evaluated using MRI. In this study, healthy mice (Group 1) were administered with Gd-DTPA; a low amount of Gd-DTPA was deposited in the brain.^145^ The second group of mice had EAE, and after administration of Gd-DTPA, a significantly higher deposition of Gd-DTPA was found in the brains compared to those in control healthy mice.^145^ This is due to the leakiness of the BBB in EAE mice. In the third group, the BPI-treated EAE mice treated were administered with Gd-DTPA and they had significantly low amounts of Gd-DTPA in the brains compared to those in untreated EAE mice.^145^ These results indicated that BPI treatments in EAE mice suppressed the disease and prevented the BBB leakiness. At the same time, BPI molecules prevented axon demyelination and CNS infiltration of immune cells.^32^

Increased levels of IL-17 have been correlated with the increase in disease activity in MS patients, where a high level of IL-17 was found in the cerebrospinal fluid and at the lesions.^68, 146^ The levels of IL-17 in the blood of Ac-PLP-BPI-NH_2_-2-treated mice were lower than in PBS-treated mice on day 35 after stimulation of the disease.^141^ PLP-BPI also enhanced the levels of TGF-β, IL-2, IL-4, IL-5, and IL-10 cytokines, indicating the stimulation of Tregs and Th2 cells.^103, 145^ BPI delivered in solution or by a controlled-release method lowered the production of inflammatory cytokines (i.e., IFN-γ, IL-6, IL-7) implying that BPI molecules prevented the proliferation of Th1 and Th17 cells.^143, 145^ In summary, BPI molecules can alter and modulate the immune cell balance between inflammatory T cells (*i.e*., Th17 and Th1) and regulatory T cells (*i.e*., Treg) to control the disease.

## 5. I-Domain Antigen Conjugate (IDAC) Molecules

A different approach to the BPI molecules is by investigating the I-domain antigen conjugate (IDAC) for the treatment of EAE. In this case, the insert-domain of the LFA-1 α-subunit (or I-domain of CD11a) was linked to several antigenic peptides to make IDAC molecules (Figure 5C; Table 2).^147–149^ The I-domain can bind to D1 of ICAM-1 on APCs.^150^ IDAC molecules were proposed to have a similar mechanism as the BPI molecules on APC (Figure 3E). Thus, this binding process prevents the formation of the IS and alters naïve T cell commitment from inflammatory to regulatory T cells (Figures 3E & 4D).^147–149^ One of the benefits of the IDAC molecules is that multiple antigenic peptides can be conjugated to the 20 lysine residues within the I-domain. Therefore, a single I-domain molecule can have several homogenic or heterogenic antigenic peptides derived from PLP, MBP, and MOG to target various EAE animal models. In other words, IDAC molecules can be conjugated with diverse antigenic peptides from PLP, MOG, and MBP for treatments of patients who are sensitive to different antigens and to prevent antigenic spreading.

IDAC molecules (i.e., IDAC-1, -2, and -3) have been shown to suppress EAE animal models as vaccines, prophylactics, or therapeutic agents.^147–149^ The lysine residues in the I-domain were modified with the maleimide groups followed by conjugation of the antigenic peptides (i.e., PLP-Cys-OH, PLP-Cys-NH_2_, and Ac-PLP-Cys-NH_2_) via a thiol group on the Cys residue. Using mass spectrometry, it was determined that each I-domain contained an average of 2.5 antigenic peptides on the IDAC molecule.^147^ The IDAC and I-domain molecules have similar circular dichroism (CD) spectra, indication that there is no secondary structural change upon conjugation of antigenic peptides to the I-domain. IDAC molecules (IDAC-1 and IDAC-3, Table 2) were evaluated in PLP-stimulated EAE by i.v. administrations (26 nmol/injection) on days 4 and 7.^147^ Another BPI molecule, called Ac-PLP-cIBR1-NH_2_ (Table 1), was administered via i.v. injections (50 nmol/injection) on days 4, 7, and 10 as a positive control. The EAE disease symptoms were significantly suppressed in animals treated with IDAC-1, IDAC-3, and Ac-PLP-cIBR1-NH_2_ compared to those treated with PBS or I-domain. IDAC-3 suppressed EAE symptoms better than IDAC-1. Two injections of IDAC-3 (26 nmol/injection) had similar efficacy as three injections of Ac-PLP-cIBR-NH_2_ (50 nmol). These results also suggest that N- and C-termini capping of the PLP peptide improves the efficacy of the IDAC-3 molecule compared to that of IDAC-1. The IDAC molecules are more potent than BPI molecules presumably due to simultaneous delivery of multiple antigenic peptides by IDAC molecules.

**Figure 6.**
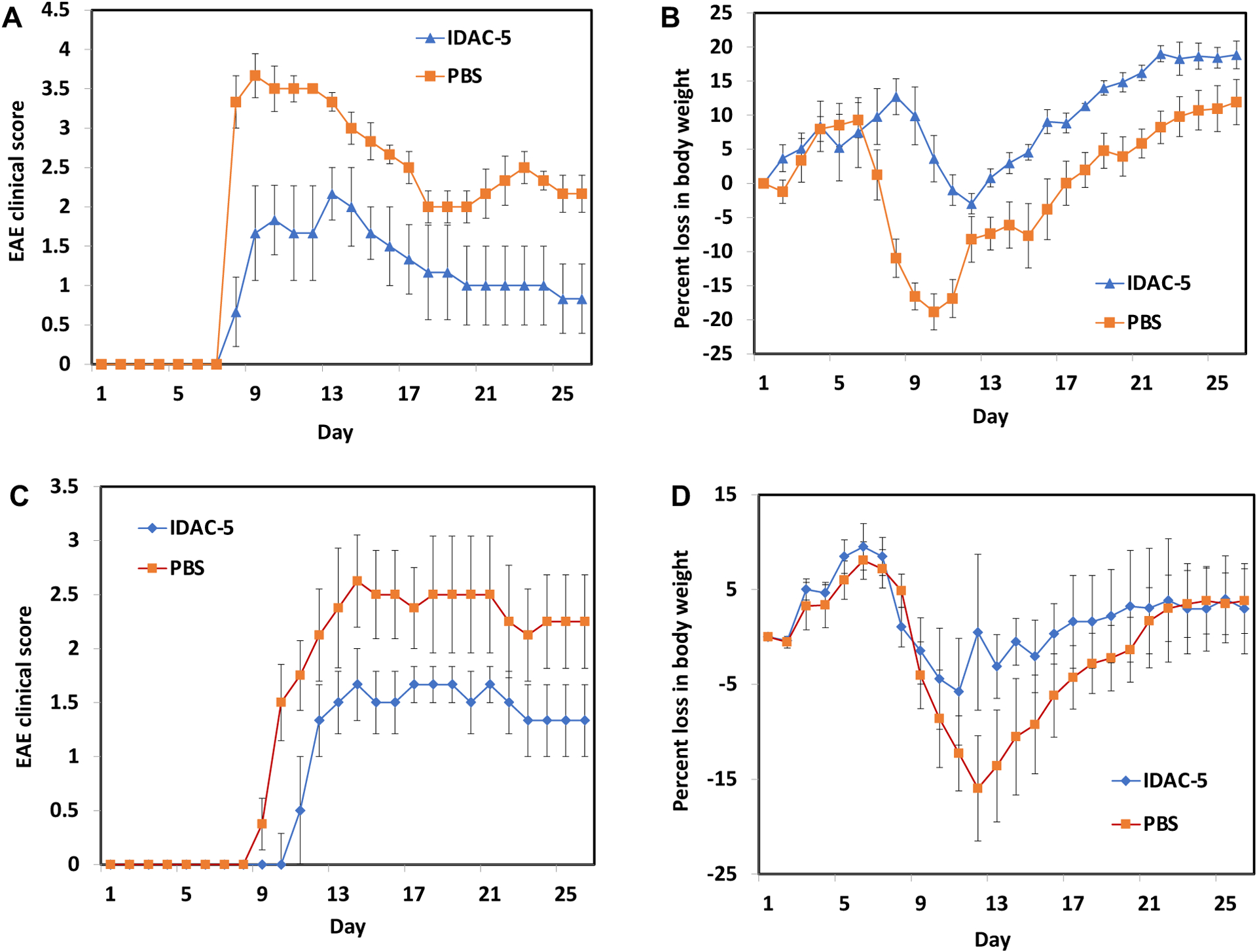
IDAC-5, containing PLP_139–151_, MBP_83–99_, and MOG_38–50_ peptides, were evaluated in suppressing EAE in PLP- and MOG-stimulated EAE using SJL/J and C57BL6 mice (n = 6), respectively. PBS-treated mice were used as a control. IDAC-5 (40 nmol) was injected once on day 4 after disease stimulation on day 0. **(A-B)** IDAC-5 suppressed the disease symptoms significantly in PLP-stimulated EAE compared to PBS as shown in **(A)** disease scores (p<0.01 between days 12–17) and **(B)** body weights. **(C-D)** IDAC-5 suppressed the disease symptoms in MOG-stimulated EAE compared to PBS as shown in **(C)** disease scores (p<0.01 between days 12–17) and **(D)** body weights.

Similarly, MOG-IDAC (IDAC-4, Table 2) was also administered on days 4 and 7 via s.c. (26 nmol/injection) to MOG-stimulated mice. As controls, EAE mice were injected with MOG_38–50_ peptide (Table 1; 100 nmol/injection) and PBS on days 4, 7 and 10. IDAC-4 suppressed both disease progression and incidence better than those injected with MOG_38–50_ peptide and PBS. Finally, IDAC-5 (Table 2, Figure 6) was synthesized to contain 3 different antigenic peptides derived from PLP, MOG, and MBP to test whether IDAC-5 could suppress both PLP- and MOG-stimulated EAE. IDAC-5 (40 nmol; n = 6) or PBS was administered once via s.c. on day 4 to PLP- and MOG-stimulated EAE mice. The results showed that IDAC-5 suppressed EAE symptoms in both PLP- and MOG-stimulated EAE mice better than PBS control (Figure 6; *p*<0.01 between days 12–17). These results suggest that IDAC molecules can be used to simultaneously deliver multiple heterogenic antigens to potentially prevent antigen spreading in EAE and MS.

IDAC-3 was administered in vaccine-like and regular treatment schedules on PLP-stimulated EAE mice.^149^ The first and second groups of healthy SJL/J mice were injected via s.c. with IDAC-3 (26 or 10 nmol/injection, respectively) on days −11, −8, and −5 prior to EAE disease induction on day 0 as a vaccine-like administration. Similarly, the third and fourth groups of SJL/J mice were treated on days −11, −8, −5 with Ac-PLP-BPI-NH_2_-2 (100 nmol/injection) as a positive control and PBS as a negative control. In the fifth group, IDAC-3 (26 nmol/injection) was injected via s.c. on days 4 and 7 after disease stimulation on day 0. When administered using a vaccine-like schedule, both IDAC-3 and Ac-PLP-BPI-NH_2_-2 significantly suppressed the EAE disease symptoms compared to the PBS-treated animals.^149^ The IDAC-3 effectiveness upon a vaccine-like administration was dose-dependent, where injections of 26 nmol IDAC-3 were more effective than 10 nmol injections. IDAC-3 administrations were more effective than Ac-PLP-BPI-NH_2_-2 and vaccine-like treatment may alter the immune cells prior to the disease induction. Therefore, further studies are needed to elucidate the mechanisms of IDAC-3 and Ac-PLP-BPI-NH_2_-2 in altering the immune cells prior to disease stimulation.

Compared to PBS treatment, IDAC-3 (26 nmol/injection) treatments significantly enhanced the IL-2 production during the peak of the disease on day 13. During the remission on day 35, the levels of IL-2 were very low in IDAC-3- and PBS-treated mice.^149^ Antigen-specific activated T cells produced IL-2 upon antigen recognition; in contrast, Treg cells suppress IL-2-producing cells to prevent T cells from self-antigen recognition.^151, 152^ Animals treated with IDAC-3 produced significantly higher levels of IL-5 but not IL-4 when compared to PBS-treated mice during the remission on day 35. Normally, Th2 and mast cells produce IL-5 for inducing B cell growth and activating eosinophils; however, overproduction of IL-5 may induce allergic reactions.^68, 83^ Higher levels of IL-10 were observed in IDAC-3-treated mice compared to those of treated with PBS. IL-10 cytokine is normally generated by T-regs, Th2, and monocytes to down-regulate inflammatory response from Th17 and Th1 cells.^33, 84^ This result was supported by suppression of IL-17, TNF-α and IFN-γ cytokines via the upregulation of Treg cells due to IDAC-3 treatments.

## 6. Fc-BPI Molecules

The Fc-BPI (Figure 5C) was designed to improve the delivery of antigenic peptides compared to BPI and IDAC molecules. The Fc-BPI is composed of the Fc-domain of an IgG1 mAb that is linked to the PLP peptide on the C-terminus while the N-terminal end is linked to the LABL peptide (Figure 5C).^7^ Since the IgG1 Fc is a homodimer, there are two N-termini and two C-termini for conjugating LABL and antigenic peptides respectively onto a single Fc molecule. The Fc region is stable on its own and remains stable with the addition of the antigenic and signal-2 blocking peptides. The advantage of the Fc-BPI compared to current IDACs is that, rather than a random mixture, the Fc-BPI is a single molecule which is easily controlled during synthesis. The molecule is more tractable analytically during preclinical and clinical studies compared to IDAC molecules. The presence of the Fc domain increases the solubility and may increase plasma stability of Fc-BPI because the Fc-domain is recognized by the FcRn receptor to improve the Fc-BPI’s circulation time in the blood stream. Because of its size, Fc-BPI avoids glomerular filtration compared to BPI molecules. Intravenous injection of the Fc-BPI containing PLP peptides on days 4 and 7 (25 nmol/injection) significantly suppressed EAE in mice compared to PBS, as determined by disease scores and body weights on days 12–17.^7^ Fc-BPI also prevented demyelination of axons in the EAE mouse brains.^7^

## 7. Antigenic and Signal-2 Peptide Conjugates to Polymers

Using the same approach as the BPI and IDAC, polymer conjugates were designed for simultaneous delivery of both antigenic peptides and signal-2 blockers to induce immune tolerance.^153–158^ Following Dintzis Rules, polymers less than 100 kDa with 1–2 antigens every 1000 Da can be tolerogenic; thus, polymers can deliver the antigen to APCs for suppressing autoreactive T cells.^157^ To test this idea, soluble antigen arrays (SAgAs) were synthesized by grafting PLP and LABL peptides on hyaluronic acid (HA) to make PLP-LABL SAgAs. Administrations of PLP-LABL SAgAs in PLP-stimulated EAE suppressed the disease; the treated mice scored significantly better than PBS-treated mice.^157^ This treatment downregulates inflammatory cytokine levels (i.e., IL-6, IL-13, IL-17, IL-22, IFN-γ, and TNF-α) while it upregulates the levels of regulatory cytokines (IL-2, IL-5).^157^

Biodegradable nanoparticles (400–500 nm) were also constructed using a combination of poly(lactic-coglycolic acid) (PLG) and poly(ethylene-co-maleic acid) (PEMA). The particles were studded with PLP_139–151_ antigenic peptides on the surface to make PLP-PLG-PEMA.^159^ Polystyrene microparticles (PLS) conjugated to PLP (PLP-PLS) were used as a positive control.^160^ As negative controls, ovalbumin peptides (OVA_323–339_) were conjugated to PLG-PEMA and PLS to produce OVA-PLG-PEMA and OVA-PLS, respectively. As expected, these negative controls did not suppress the EAE disease symptoms. Both PLP-PLG-PEMA and PLP-PLS were very effective in suppressing EAE; however, PLP-PLG-PEMA was better than PLP-PLS. As a vaccine-like treatment, administrations of PLP-PLG-PEMA on −7 or −25 day before stimulation of the disease on day 0 can suppress the EAE disease symptoms as well as production of IL-17 and IFN-γ cytokines. These treatments also reduced the infiltration of Th17 and Th1 cells into the CNS. Later, the effect of a combination of PLG and poly(DL-lactide) (PLA) nanoparticles to deliver PLP_139–151_ peptide in EAE was investigated. In this case, PLA induced the APC to be more tolerogenic. In addition, PLA increases nanoparticles deposition in the liver to improve the lasting impact of nanoparticles on immune tolerance.^161^

## 8. Antigenic Peptide-Drug Conjugates

Peptide-drug conjugates are another class of drugs that aim to specifically target immunomodulatory agents against diseased cells in autoimmune diseases.^162^ The PLP antigenic peptide was conjugated to dexamethasone (Dex) to make a PLP-Dex conjugate that was evaluated for its ability to suppress EAE in mice.^162^ Dexamethasone effectively suppressed proinflammatory cytokines in the EAE mice to counteract the costimulatory signal of B7/CD28. The conjugate induced the upregulation of CTLA-4 to reduce a proinflammatory response.^162^ While effective, dexamethasone can lead to general immune suppression; therefore, the addition of the antigenic peptide helps its targeted delivery to a subpopulation of immune cells. PLP-Dex can effectively suppress EAE in mice compared to those treated with dexamethasone alone or mannitol.^162^ The results suggest that a combination of antigenic peptide and drug together can suppress the disease in an antigen-specific manner.

## 9. Mechanisms of BPI and BPI Related Molecules

In all previous studies, BPI molecules are more potent than the respective antigenic peptides alone. It is proposed that BPI molecules induce production of Treg cells and suppress inflammatory T cell proliferation through three different mechanisms while the antigenic peptides work via one mechanism (Figure 4A). In the first mechanism, BPIs work in the same manner as soluble antigenic peptides (Figure 4A). In this case, the BPI binds to the surface of the immature dendritic cell (iDC) via empty MHC-II through docking of the antigenic peptide portion of the BPI.^60, 63, 66, 163^ Because iDC does not express CD80/CD86, binding of naïve T cell to iDC via TCR/Ag-MHC-II complex in the absence of CD28 binding to CD80/CD86 results in naïve T cell differentiation to Treg cells. In the second mechanism, the antigenic peptide fragment of the BPI binds to the empty MHC-II while LABL binds to ICAM-1 on the surface of the iDC (Figure 4B). Because of the additional binding of LABL peptide on BPI to ICAM-1, the population of BPI is higher on iDC compared to antigenic peptide alone. Furthermore, binding of LABL fragment blocks the LFA-1/ICAM-1 costimulatory signal as well as the absence of CD28/CD80/86 costimulatory signal. Therefore, the production of Treg cells is more pronounced in mice treated with BPI compared to those treated with antigenic peptide alone. In inflammatory conditions, naïve T cells can also binds to mature DC (mDC) by forming immunological synapse via TCR/MHC-II and LFA-1/ICAM-1 or CD28/CD80/86 to generate inflammatory Th17 and Th1 cells (Figure 4C). Thus, the third potential mechanism of BPI is that it interacts with mDC in which BPI molecule simultaneously binds to MHC-II and ICAM-1 on the surface of mDC (Figure 4D). Upon mDC interaction with T cell, there is a connection between TCR and Ag-MHC-II complex; however, because LABL fragment binds to ICAM-1, it blocks of the binding between LFA-1 and ICAM-1 as costimulatory signal. Thus, the IS formation is inhibited to generate anergic effects on T cell activation.^6^ Therefore, naïve T cells do not undergo clonal expansion to produce inflammatory Th1 and Th17 cells (Figure 4D). In summary, it is proposed that the superior efficacy of BPI molecules over antigenic peptides is due three different mechanisms of BPI activity. Overall, treatments with BPI molecules and antigenic peptides tip the balance of immune cells from inflammatory to regulatory response followed by the restoration of the immune system balance to normal conditions.

## 10. Conclusion

Autoimmune diseases are difficult to treat due to their autoreactive nature. Many therapies help reduce the intensity of the disease, but often can suppress the general immune system. This shortcoming makes patients susceptible to opportunistic infections. To account for this limitation, antigen-specific immunotherapies are being developed to selectively target the diseased cell population while leaving the general immune system uncompromised. Soluble antigenic peptides and peptide conjugates (BPI, IDAC, Fc-BPI, polymer conjugates, and peptide-drug conjugates) have shown good-to-excellent efficacies in suppressing the EAE disease. BPIs and antigenic peptides are able to suppress the disease by binding to APCs to modulate the differentiation and proliferation of a specific subpopulation of T cells to a regulatory response rather than an inflammatory response. Some encouraging results have shown that antigenic peptides and BPI molecules can be used as prophylactics, treatments, and vaccines to suppress autoimmune diseases and alter the balance of immune cells to a regulatory phenotype. Although these treatments have been effective in animal models of MS, the mechanisms of action and potential side effects of these antigen-specific therapeutics still need further investigation. In the future, their mechanisms of action will be used to improve their efficacy and safety in treating MS and other autoimmune diseases patients.

## Figures and Tables

**Table 1.** The Sequences of Antigenic Peptides, BPI, and Multivalent BPI

Peptides	Sequences
GAD_208–217_	EIAPVFVLLE
GAD-BPI	EIAPVFVLLE-AcpGAcpGAcp-ITDGEATDSG
MOG_38–50_	GWYRSPFSRVVHL
MBP_83–99_	DENPVVHFFKNIVTPRT
PLP_139–151_ or PLP	HSLGKWLGHPDKF
PLP-BPI	HSLGKWLGHPDKF-*AcpGAcpGAcp*-ITDGEATDSG
OVA-BPI	AVHAAHAEINEA-*AcpGAcpGAcp*-ITDGEATDSG
LABL	ITDGEATDSG
Ac-PLP-cIBR1-NH_2_	Ac-HSLGKWLGHPDKF-(AcpGAcpGAcp)_2_-Cyclo(1,12)-PenPRGGSVLVTGC-NH_2_
PLP_L,R_ (LR-APL)	HSLGKLLGRPDKF
Ac-PLP-BPI-PEG3	Ac -HSLGKWLGHPDKF-Acp-(C_2_H_5_O)_3_-Acp-ITDGEATDSG-NH_2_
Ac-PLP-BPI-PEG6	Ac-HSLGKWLGHPDKF-(C_2_H_5_O)_3_-G-(C_2_H_5_O)_3_-ITDGEATDSG-NH_2_
Ac-PLP-BPI-NH_2_-2	Ac-HSLGKWLGHPDKF-(*AcpGAcpGAcp*)_2_-ITDGEATDSG-NH_2_
Ac-LABL-PLP-NH_2_	Ac-ITDGEATDSG-*AcpGAcpGAcp*-HSLGKWLGHPDKF-NH_2_
MOG-BPI	Ac-GWYRSPFSRVVHL-XGX-ITDGEATDSG-NH_2_
MVB_MOG/PLP_	Ac-GWYRSPFSRVVHL-XGX-ITDGEATDSG-XGX-HSLGKWLGHPDKF-NH_2_

Acp = Aminocaproic acid; X = polyethyleneglycol-3 (PEG3) or -(C_2_H_5_O)_3_-; G = Gly

**Table 2.** Structures of IDAC Molecules

IDAC	Conjugate Sequences
GMB-I-domain	[*N*-(γ-maleimido)-1-oxybutyl]_n_-I-domain
IDAC-1	(HSLGKWLGHPDKFC)_n_-linker-I-domain
IDAC-2	(HSLGKWLGHPDKFC)_n_-linker-I-domain
IDAC-3	(Ac-HSLGKWLGHPDKFC-NH_2_)_n_-linker-I-domain
IDAC-4	(Ac-GWYRSPFSRVVHLC-NH_2_)_n_-linker-I-domain
IDAC-5 (PLP-MOG-MBP-IDAC)	(Ac-HSLGKWLGHPDKFC-NH_2_)_n_-linker------_I_ (Ac-ASQKRPSQRSKC-NH_2_)_n_-linker----**I-domain** (Ac-GWYRSPFSRVVHLC-NH_2_)_n_-linker------^I^

Linker = *N*-(γ-maleimido)-1-oxybutyl

## Abbreviations:

APL: altered peptide ligands
Ag-MHC-II: antigen-MHC-II complexes
APC: antigen presenting cell
BPI: bifunctional peptide inhibitor
BBB: blood-brain barrier
CNS: central nervous system
cSMAC: central supramolecular activation complex
CII: collagen II
CIA: collagen-induced arthritis
CFA: complete Freund’s adjuvant
DTH: delayed-type hypersensitivity
DCs: dendritic cells
DMARDS: disease-modifying antirheumatic drugs
Dex: dexamethasone
DMTs: disease modifying therapies
D1: domain 1
EAE: experimental autoimmune encephalomyelitis
Fc-BPI: Fc-bifunctional peptide inhibitors
Gd-DTPA: gadolinium diethylene-triamine-pentaacetate
GAD: glutamic acid decarboxylase
HA: hyaluronic acid
IDAC: I-domain antigen conjugates
iDCs: immature dendritic cells
IS: immunological synapse
ICAM-1: intercellular adhesion molecule-1
IA-2: insulinoma antigen-2
IFN-γ: interferon gamma
IL: interleukin
LFA-1: leukocyte function associated antigen-1
MRI: magnetic resonance imaging
MHC: major histocompatibility complex
mAbs: monoclonal antibodies
MS: multiple sclerosis
MBP: myelin basic protein
NOD: non-obese diabetes
MOG: oligodendrocyte glycoprotein
OVA: ovalbumin
PRR: pattern recognition receptors
pSMAC: peripheral supramolecular activation complex
PAD: peptidyl arginine deiminases
PBS: phosphate buffer saline
PEMA: poly(ethylene-co-maleic acid)
PLA: poly(DL-lactide)
PLG: poly(lactic-coglycolic acid)
PLGA: poly(D,L-lactic-co-glycolic acid)
PLS: polystyrene microparticles
PPMS: primary progressive MS
PML: progressive multifocal leukoencephalopathy
PRMS: progressive-relapsing MS
PLP: proteolipid protein
Treg: regulatory T cell
RRMS: relapsing-remitting MS
RA: rheumatoid arthritis
SPMS: secondary progressive MS
SEC: size-exclusion chromatography
SAgAs: soluble antigen arrays
s.c.: subcutaneous
TCR: T-cell antigen receptors
Th1: T helper-1
Th17: T helper-17
TGF-β: transforming growth factor beta
TG2: transglutaminase 2
TNF-α: tumor necrosis factor alpha
T1D: Type-1 diabetes
VCAM-1: vascular cell adhesion molecule-1
ZNT8: zinc transporter 8
